# Flexible 3D printed microwires and 3D microelectrodes for heart-on-a-chip engineering

**DOI:** 10.1088/1758-5090/acd8f4

**Published:** 2023-06-22

**Authors:** Qinghua Wu, Peikai Zhang, Gerard O’Leary, Yimu Zhao, Yinghao Xu, Naimeh Rafatian, Sargol Okhovatian, Shira Landau, Taufik A Valiante, Jadranka Travas-Sejdic, Milica Radisic

**Affiliations:** 1 Institute of Biomedical Engineering, University of Toronto, Toronto, ON M5S 3G9, Canada; 2 Department of Chemical Engineering and Applied Chemistry, University of Toronto, Toronto, ON M5S 3E5, Canada; 3 School of Chemical Sciences, The University of Auckland, Auckland 1010, New Zealand; 4 MacDiarmid Institute for Advanced Materials and Nanotechnology, Wellington 6140, New Zealand; 5 Krembil Brain Institute, University Health Network, Toronto, ON M5G 2C4, Canada; 6 Toronto General Research Institute, University Health Network, Toronto, ON M5G 2C4, Canada; 7 Department of Chemical Engineering, Polytechnique Montreal, Montreal, QC H3T 1J4, Canada; 8 The Edward S. Rogers Sr. Department of Electrical and Computer Engineering, University of Toronto, Toronto, ON M5S 3G4, Canada; 9 Department of Surgery, Division of Neurosurgery, University of Toronto, Toronto, ON M5T 1P5, Canada; 10 Terrence Donnelly Centre for Cellular & Biomolecular Research, University of Toronto, Toronto, ON M5S 3E9, Canada

**Keywords:** PEDOT:PSS, 3D microelectrodes, 3D printing, cardiomyocytes, heart-on-a-chip, force sensor, extracellular field potential

## Abstract

We developed a heart-on-a-chip platform that integrates highly flexible, vertical, 3D micropillar electrodes for electrophysiological recording and elastic microwires for the tissue’s contractile force assessment. The high aspect ratio microelectrodes were 3D-printed into the device using a conductive polymer, poly(3,4-ethylenedioxythiophene):poly(styrene sulfonate) (PEDOT:PSS). A pair of flexible, quantum dots/thermoplastic elastomer nanocomposite microwires were 3D printed to anchor the tissue and enable continuous contractile force assessment. The 3D microelectrodes and flexible microwires enabled unobstructed human iPSC-based cardiac tissue formation and contraction, suspended above the device surface, under both spontaneous beating and upon pacing with a separate set of integrated carbon electrodes. Recording of extracellular field potentials using the PEDOT:PSS micropillars was demonstrated with and without epinephrine as a model drug, non-invasively, along with *in situ* monitoring of tissue contractile properties and calcium transients. Uniquely, the platform provides integrated profiling of electrical and contractile tissue properties, which is critical for proper evaluation of complex, mechanically and electrically active tissues, such as the heart muscle under both physiological and pathological conditions.

## 1. Introduction

The organ-on-a-chip field relies on engineered, microfabricated devices to replicate and measure critical physiological properties (e.g. contractility), that are required for both drug discovery and disease modeling [1–3]. When focusing on the heart, which is both a physically contractile and electrically active organ, the contractile forces and electrophysiology of the tissue are essential for understanding functional development and disease manifestations [4]. In standard 2D cardiomyocyte (CM) cultures, beating properties can be estimated from impedance measurements on gold-coated plates [5, 6]. Additionally, 2D microelectrode arrays (MEAs) are routinely used for extracellular field potential recordings [7, 8]. Yet, iPSC-derived CMs in standard 2D cultures often suffer from poor maturation [9, 10]. On rigid substrates, MEAs alter the natural mechanical environment of the tissue and preclude accurate recording of the contractile force in the same setup used for electrophysiological recordings. These limitations motivate the use of a suspended 3D tissue [11].

In 3D culture, the motion of cardiac tissue is often used to assess contractile dynamics, without precise enumeration of contractile force or stress [12–15]. Similarly, in polydimethylsiloxane (PDMS)-based microfluidic devices and stencilled dog-bone cardiac tissues, contractile dynamic, such as beating rate, is routinely assessed by optical analysis of cardiac tissue displacement [16–19]. A number of studies have described a more precise enumeration of the cardiac tissue contraction force by measuring the deflection of structures of known elasticity and stiffness, such as PDMS posts [20], PDMS cantilevers [21] or thin films [22, 23]. The contractile force of cardiac tissues can also be assessed by calibrated flexible probes [24]. We have previously used manually produced elastomeric polymer wires as displacement sensors in a biowire II device [11], to calculate contraction force via force-displacement calibration curves.

Simultaneous recording of contractile force and electrophysiological measures has proven more difficult for 3D tissues. Pioneering studies reported planar microelectrodes and MEAs integrated within cardiac patches [25], wrapped around cardiac organoids [26], integrated into nanotopographical substrates [27, 28] or microfluidic devices [29] to enable electrical recording from cardiac tissues. Yet, contractile force assessment in the same setup remains elusive. In an advanced 3D printed device, PDMS cantilevers [30] were instrumented with planar electrodes to record tissues a few cell layers in thickness. While this technique enables simultaneous force and electrical recordings, recording from thicker tissues is not possible, due to the planar electrode configuration.

Recent reports have demonstrated that 3D electrodes, typically vertical micro/nano-pillars, can detect extracellular potentials [31–33]. Metals or metal-like materials such as iridium oxide [33], platinum [32] and gold [34], are commonly used to fabricate such electrodes. However, the elastic modulus of these materials is five orders of magnitude higher than that of cells and the extracellular matrix, resulting in an extremely mechanically mismatched cell-electrode interface. Stiff electrodes could cause tissue damage over longer periods (weeks) of contractile activity.

In contrast, an organic conducting polymer, poly(3,4-ethylene dioxythiophene) doped with polystyrene sulfonate (PEDOT:PSS) promises to solve these problems due to its marked biocompatibility, high flexibility and conductivity [35]. Its current applications include wearable and implantable sensors for electrophysiological signals or biological markers [36, 37], electrical stimulation electrodes [38], scaffolds for tissue engineering and drug release systems [39]. Recent pioneering studies reported on PEDOT:PSS 2D arrays [40, 41] and 3D electrodes [42] designed to measure electrophysiological properties [43]. We recently developed a rapid and simple ‘direct writing’ process for 3D printing of PEDOT:PSS MEAs [35] and applied it for the three-dimensional electrical stimulation of neurons derived from human neural stem cells into maturing neural tissues [41]. Yet, neural tissues are not mechanically active, thus 3D printed electrodes were always stationary without stringent mechanical requirements.

**Figure 1.**
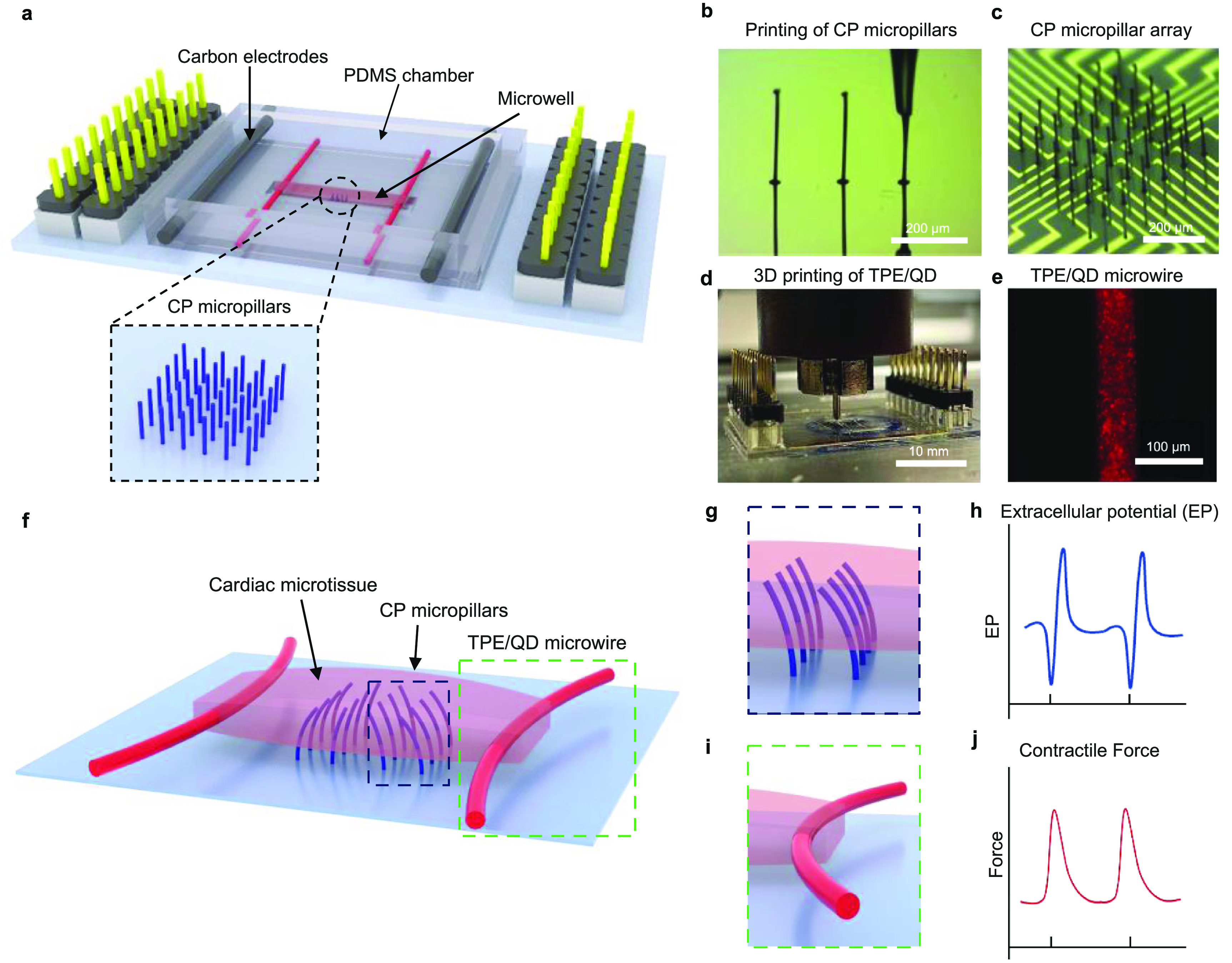
A heart-on-a-chip platform integrating stimulating and recording electrodes with force sensors. (a) Schematic illustration of the device consisting of a soft conductive polymer (CP) micropillar array (blue) for extracellular potential recording, TPE/QD nanocomposite microwire (red) for force sensing, a microwell for seeding cardiac tissue and carbon electrodes (dark grey) for electrical stimulation of cardiac tissue. (b) An optical image showing direct writing of a CP micropillar (scale bar, 100 *μ*m). (c) A microscopy image of the CP micropillar array (scale bar, 200 *μ*m). (d) An optical image illustrating 3D printing of TPE/QD nanocomposites on both sides of the microwell (scale bar, 10 mm). (e) A representative fluorescent image of the nanocomposite microwire (scale bar, 100 *μ*m). (f) Schematic illustration of the cardiac microtissue generated from the device, showing the CP micropillars embedded in the tissue and the TPE/QD microwires deflected by the cardiac microtissue. (g) Schematic illustration of the 3D CP micropillars in the device and (h) schematic illustration of the extracellular potential detected by the CP micropillars. (i) Schematic illustration of the TPE/QD microwire used as a force sensor. Microwire bending due to pre-tension of the cardiac microtissue. (j) Schematic illustration of the contractile force measured by fluorescent tracing of the microwires.

Here, we developed a new heart-on-a-chip platform with built-in multimaterial 3D microstructures for non-invasive *in situ* acquisition of electrophysiological and contractile functional readouts (figure 1). The platform integrated vertical 3D PEDOT:PSS microelectrodes for electrophysiological sensing, with built-in thermoplastic elastomer (**TPE**) and quantum dot (**QD**) nanocomposite microwires, that acted as both tissue anchor points and displacement sensors for evaluation of contraction force. To critically improve the mechanical and electrical coupling at the tissue/microstructure interface and enable recording from 3D tissues, the elastic modulus and stiffness of both the micropillar electrodes and nanocomposite microwires were specifically tailored to match those of cardiac tissues. The new device enables profiling of a multitude of functional properties of the cardiac tissue with and without epinephrine as a model drug: specifically, extracellular field potentials and contractile force, as well as calcium transients when combined with standard fluorescence microscopy and Ca^2+^ sensitive dyes. Additionally, the device provides a capability for continuous external electrical field stimulation via a pair of separately integrated carbon electrodes.

## 2. Materials and methods

### 2.1. Fabrication of PEDOT:PSS pillar arrays

Au electrode arrays were prepared from Au and Ticoated glass slides using the photolithography technique with patterned SU-8 (MicroChem Corp) as the insulator layer. The detailed fabrication process was described in our previous work **[** 35 **]**. Each working electrode array was comprised of 36 individually addressable microelectrodes in a 6 × 6 array format. The top insulating layer had 20 *µ*m diameter openings to expose the corresponding areas of the underlying patterned Au electrodes where the conductive polymer (CP) pillars were printed. The distance between the electrodes was 100 *µ*m. Each Au electrode was linked to a larger square connection pad, which was then connected via a pin connector, to the electrochemical workstation (Biologic SP-300).

The micropillars (PEDOT:PSS) were fabricated on micro-electrode Au arrays as previously reported by us [35, 41]. Briefly, a thin layer of PEDOT:PSS film was electrochemically polymerized onto the Au microelectrodes to diminish the contact resistance and also to enhance the bonding between the CP pillar and the Au electrodes. This was followed by the 3D writing of CP microelectrodes using a solution of PEDOT:PSS (CLEVIOS™ PH 1000), dimethyl sulfoxide and the (3-glycidyloxypropyl)trimethoxysilane (GOPS) crosslinker (Sigma-Aldrich) as the ‘ink’. The ink was injected by a MicroFil (World Precision Instruments) into a micropipette, which was fabricated from a single barrel borosilicate capillary using a laser puller P-2000 (Sutter Instrument). The diameter of the micropipette tip was about the same diameter as the Au microelectrodes. The PEDOT ink-filled micropipette was precisely positioned over the Au electrode using a home-constructed printing system [44]. Once the meniscus of the ‘ink’ at the tip of the pipette established contact with the substrate, the micropipette was raised by 1.5 *µ*m s^−1^ using a programme with LabVIEW. The pipette was pulled up at a speed that allowed for the evaporation of the solvents in the ink and formation of the CP microelectrodes (figure 1(b)). The samples were placed on a hot plate (30 °C) during the printing to promote evaporation of solvents.

### 2.2. Fabrication of nanocomposite microwires

A nanocomposite ink was prepared by mixing core–shell CdSe/ZnS QDs (stabilized with octadecylamine ligands, fluorescence *λ*_em_ 630 nm, from Sigma-Aldrich) and poly(styrene) (ethylene/butylene)–(styrene) copolymer TPE (Versaflex CL30, lot 5112 553, Viscosity of 14 800 cP at shear rate of 11 200 S^−1^ at temperature of 200 °C, PolyOne). Briefly, TPE (10 g) was mixed with QDs (5 mg) in toluene (20 ml, from Sigma-Aldrich) to obtain a concentration of 0.05 w/w% QDs in the polymer. After solvent evaporation overnight at room temperature in a fume hood, the elastomer-based material was baked in an oven under a vacuum at 75 °C, for 1 h. The resulting thermoplastic-based nanocomposite ink was loaded into a temperature-controlled chamber of a 3D bioprinter (RegenHU Ltd, Switzerland) and then deposited on a three-axis positioning platform via a 60 *µ*m micronozzle at 210 °C under an applied pressure of 0.05 MPa with a robot velocity of 1 mm s^−1^ (figure 1(d)).

### 2.3. Fabrication of the heart-on-a-chip device

The MEA arrays with 250 (±10) *μ*m micropillars were prepared as described above. A custom sheet including a bottomless cardiac tissue microwell (5 × 1 × 0.3 mm^3^, length (*L*) *×*width (*W*) × thickness (*H*)) was prepared from polydimethylsiloxane (PDMS, Sylgard 184 silicone elastomer kit, 1:10 ratio of crosslinker to elastomer) using casing on a SU-8 photoresist master fabricated by standard soft lithography and placed on top of the MEA, such that the rectangular well in the center accommodated the micropillar electrodes. The nanocomposite microwires were directly deposited on top of the PDMS microwell. Two carbon electrodes (1/8’ diameter, Ladd Research Industries) were placed in such a way that the long axis of the microwell was perpendicular to the electrodes. This would ensure that the developing cardiac tissue orients along the field lines. The carbon electrodes were connected via platinum wires (0.004” diameter, Ladd Research Industries) to an external stimulator. After 3D printing of TPE/QD nanocomposite microwires, a bottomless PDMS chamber (20 × 18 × 12 mm^3^, *L × W × H*) was adhered to the device using uncrosslinked PDMS. The device was adhered to a Petri dish (Fisher Scientific) for cell culture using a polyurethane adhesive (SP 1552–2, GS Polymers, Inc.). The entire set-up was then sterilized with ethylene oxide (Medical Device Reprocessing Department, Toronto General Hospital). Upon sterilization, the device was used for cell culture. Prior to electrophysiological measurements, the electrode pads on the edges of the conductive polymer micropillars (200 *µ*m) were connected to a custom printed circuit board (PCB) with electrophysiological amplification and digitization circuits (Intan RHS2116, Intan Technologies). The board was connected to a digital headstage controller (RHS Stim/Recording Controller, Intan Technologies).

To assess the effects of tissue remodeling on the electrodes, the distance between two micropillars in the topmost row of the array was measured at day 1, 3, and 7 after cell seeding. The values were normalized to the distance between the two Au electrodes on the base. Additionally, the projection of micropillar length in the topmost row of the array was measured at day 1, 3, and 7 after cell seeding.

### 2.4. Electrochemical characterization of the PEDOT:PSS microelectrodes

Cyclic voltammogram (CV) and electrochemical impedance spectra (EIS) measurements of CP microelectrodes of different heights (50–250 *μ*m) and of electrochemically deposited PEDOT films (without microelectrodes) were performed. CV measurements were performed in PBS buffer and scanned from −0.9 to 0.6 V (*vs.* Ag/AgCl), at a scan rate of 100 mV s^−1^. The EIS was performed in the presence of 5 mm of Fe(CN)_6_^3-/4-^ (in PBS) using a sinusoidal excitation signal of 10 mV in a frequency range of 1 Hz and 7 MHz.

### 2.5. Mechanical characterization of the PEDOT:PSS microelectrodes

PEDOT:PSS microelectrodes force-displacement was measured using a microscale mechanical tester (MicroSquisher, CellScale). A 0.15 mm diameter cylindrical probe was used to bend the microelectrodes at a speed of 1 *µ*m s^−1^ while the force was recorded simultaneously (figure S4). The measurements were first carried out under dry conditions, in air and at room temperature and then in culture media at 37 °C, after a 10 d incubation in customized Induction 3 Medium (I3M) (StemPro-34 complete media, 1% GlutaMAX, 20 mm HEPES, 1% Penicillin-Streptomycin, Life Technologies; 150 *μ*g ml^−1^ transferrin, 213 *μ*g ml^−1^ 2-phosphate Ascorbic Acid, Sigma-Aldrich). During measurements, the electrodes were fully submerged. The customized configuration of the CellScale instrument, which enables the mounting of well plates, allowing for facile measurements with the probe reproducibly placed at the tip of the microelectrode. The force, probe displacement (0–50 *µ*m) and time were recorded (*n* > 10). A simple linear regression analysis was performed to calculate the slope for each tested sample.

The elastic modulus of the pillar, *E*, was calculated using the following equation: $E = \frac{{P{L^3}}}{{3WI}}$, where *P* represents a point load applied on the microelectrode, *W* is the vertical displacement of the pillar at the contact point between the circular probe and the micropillar, *L* is the height of the pillar and *I* represents a moment of inertia that is given by the equation: I = π/4* radius^4^.

The **stiffness (*K*)** of the pillars was calculated by the equation of $K = \frac{P}{W}$, which can be calculated from the elastic modulus by the equation of $\frac{P}{W} = \frac{{3EI}}{{{L^3}}}$.

### 2.6. Tensile testing of TPE/QD nanocomposites

The tensile properties of QD/TPE nanocomposites were measured using dog-bone-shaped slabs. As per ASTM D638-14 standard, dog-bone samples (width of 5 mm and thickness of 3 mm) were obtained by injection molding (DSM IM5.5, DSM Netherlands) the TPE/QD nanocomposite material into a metal mold. The slabs of the nanocomposite samples were immersed in I3M media for 24 h, one week and one month. For each time point, the weight and Young’s moduli were compared to the actual slabs before immersion for the indicated time period, in order to minimize the effects of sample-to-sample variability. The weight change of those samples was measured before and after culture media immersion, followed by tensile testing. Tensile testing was performed using an Electroforce 5200 Biodynamic Test Instrument (BOSE), under a strain rate of 0.1 mm s^−1^, to an ultimate strain of 120%. The force-displacement data was collected using WinTest software. Young’s modulus was calculated by using stress–strain data from the first 20% of strain.

### 2.7. Force-displacement curves of TPE/QD nanocomposite microwires

The force–displacement curves of TPE/QD microwires were measured by using a microscale mechanical tester (MicroSquisher, CellScale). Customized tips, (half ellipse, 4:1 diameter ratio) generated from a SU-8 mold by soft lithography, of 500 *μ*m, 700 *μ*m, and 800 *μ*m (long diameter of the half ellipse), were adhered to a tungsten probe (0.1524 mm) by using an adhesive (T-GSG-01 Titan Gel), to recapitulate the curvature and diameter of tissues on the nanocomposite wires. The probe was used to bend the nanocomposite wire perpendicularly from the middle point at a velocity of 2.5 *μ*m s^−1^ (figures 3(f), (h) and (i)). To test for stability and since it is difficult to remove the tissue from the microwire after cultivation without destroying the microwire, the nanocomposite microwires were incubated in culture media at 37 °C for 6 weeks. Subsequently, they were tested for force-displacement using a 500 *μ*m customized tip. The probe displacement and force were recorded for generating force-displacement calibration curves. The calibration curves using different customized tips were calculated to be:

\begin{align*} &amp; y = 1.86 \times 10^{-5} x^3 + 0.0021x^2 + 0.50x\; (500 \mu \text{m-tip}),\nonumber\\[6pt] &amp; y = 4.66 \times 10^{-5} x^3 + 0.0035x^2 + 0.54x\; (700 \mu \text{m-tip}),\nonumber\\[6pt] &amp; \text{and }\nonumber\\[6pt] &amp; y = 1.61 \times 10^{-5} x^3 + 0.0057x^2 + 0.20x\; (800 \mu \text{m-tip}) \end{align*}

where y is the force in *μ*N, and *x* is the displacement in *μ*m. Prism 9.0 was used for data analysis and generating fitted curves, 95% confidence interval curves and *R* ^2^ values. The contraction force of the tissue was calculated by interpolation of the above calibration curves, based on the measurement of tissue widths on the nanocomposite wire.

### 2.8. Transmission electron microscopy

To characterize QDs in QD/TPE nanocomposite using TEM, 40 *µ*L of QD/TPE suspension was pipetted to 200 mesh copper grids (electron microscopy sciences). The grids were then stained with saturated uranyl acetate (2.5%) for 5 min, followed by DI water wash. Grids were washed three times by depositing 5 *µ*l of DI water and wicking. Negative staining was performed by adding 5 *µ*l of 2% uranyl acetate to grids followed by 30 s of incubation and wicking. Images were acquired at 22 000x, 45 000x, and 92 000x on a Talos L120C TEM (Thermo-Fisher Scientific).

### 2.9. Generation of engineered cardiac tissues

Human iPSC CMs were obtained from hiPSC line BJ1D (a kind gift from Dr William Stanford) [11] using monolayer differentiation protocols [45, 46]. On days 18–21 of stem cell differentiation, CMs were disassociated into single cells using previously described methods [46]. The disassociated cells were mixed with cardiac fibroblasts (Lonza, NHCF-V) at a ratio of 10:1 and then mixed in a collagen-based hydrogel at a cell density of 6 × 10^7^ cells ml^−1^. The collagen hydrogel (500 *µ*l) was formed by mixing rat tail collagen (153 *µ*l at 9.82 mg ml^−1^, Corning), 1X M199 (50 *µ*l, Sigma), Matrigel (75 *µ*l, BD Biosciences), deionized sterile H_2_O (167 *µ*l), NaOH (5 *µ*l at 1 M, Sigma) and NaHCO_3_ (50 *µ*l at 2.2 mg/ml, Sigma). The cell-laden hydrogel (2 *µ*l per microwell) was seeded in the microwell of the device chamber. After placing 1 ml medium on the outside of the device in the Petri dish to maintain moisture, the Petri dish was incubated at 37 °C, 5% CO_2_ for 10 min to allow for hydrogel gelation, after which, 5 ml of cell culture was added to the device chamber. The cardiac tissues were then incubated in I3M at 37 °C, 5% CO_2_ for 7 d for the majority of measurements demonstrated in this manuscript. The culture medium was changed twice a week. Three tissues were cultivated for up to five weeks. The tissue morphology was observed daily using an Olympus CKX41 inverted microscope.

### 2.10. Cytotoxicity assessment

Cytotoxicity was assessed by conditioning the culture media with the TPE/QD nanocomposite to collect the leachates for up to one month and then applying the conditioned media to fibroblasts according to our previous method **[** 47 **]** and consistent with ISO 10 993-5:2009 standards. We used a lactate dehydrogenate Cytotoxicity Assay Kit (Cayman Chemical). TPE/QD nanocomposite samples were immersed in Cardiac Fibroblast Growth Medium-3 BulletKit™ (Life Technology) for up one month in the incubator (37 °C) to generate conditioned media. To generate the samples, the pieces of the nanocomposite were pressed via a hydraulic press (Carver Press Manual Bench Press) at 175 °C for 5 min to form a uniform slab. The created slab was then cut into films with a dimension of 5 mm wide × 20 mm long × 1 mm thick. Conditioned media was prepared by immersing composite films in media, with a surface-to-liquid ratio of 6 cm^2^ ml^−1^ according to the Canadian Standards Association. Nanocomposite films were immersed in fibroblast media 3 (PromoCell, 2020) and incubated at 37 °C for up to 1 month, with three replicates for each time point. Devices with and without micropillars were incubated with culture media for up to two weeks for cytotoxicity assessment.

Human cardiac fibroblasts (Clonetics™ NHCF-V, LONZA) were seeded in a 24-well plate (Corning) and allowed cell attachment overnight. Cells were then treated with dilutions of conditioned media (1×, 2×, 5×, 10×) in Cardiac Fibroblast Growth Medium-3 BulletKit™ (Life Technology) and culture medium from the devices, separately. Cell viability was quantified and compared against untreated (negative) control as well as a positive control (supernatant collected from CMs treated with 1% Triton-X for 24 h) per the manufacturer’s instructions of the kit. Cytotoxicity was assessed by the Lactate dehydrogenase (LDH) release from culture media using a commercially available kit (Cayman Chemical). A calibration curve was constructed to correlate LDH release to known values of fibroblast cell death of positive control.

### 2.11. Calcium transient recording and analysis

Cardiac tissues in the devices were incubated with the Ca^2+^ dye fluo-4 NW (Thermo Fisher) for 40 min, at 37 °C. Ca^2+^ transients before and after the addition of epinephrine (10 *μ*m), as previously described [48], were recorded under a fluorescence microscope, using a green-light channel (*λ*_ex_490 nm/*λ*_em_525 nm). The ratio of peak fluorescence to baseline fluorescence intensity was measured by the relative Ca^2+^ change in the tissues by Image J software (NIH), as described previously [11]. Prism 9.0 was used for all the calculations and for the generation of all figure plots.

### 2.12. Contractile force recording and analysis

The contractile behavior of cardiac tissues was recorded by placing the heart-on-a-chip set-up in the environmental chamber (37 °C, 5% CO_2_) of the fluorescence microscope (Olympus CKX41 inverted microscope). The minimum voltage per cm required to cause synchronized contraction of the cardiac tissue (excitation threshold (ET)) was determined by observing tissue displacement via an optical microscope under electrical stimulation at 1 Hz provided by an external stimulator (Grass Technology S88X Square Pulse Stimulator) by using carbon electrodes (10 mm spacing between two electrodes) on both sides of the cardiac tissue. Biphasic stimulation (1 ms duration per pulse) was used to stimulate the tissues. The maximum capture rate (MCR) of the tissue under synchronized beating, in response to the electrical stimulation at a voltage of twice the ET, was measured as the maximum frequency at which tissues lost synchronized contraction. The readouts of the contractile dynamics including active force, passive tension, and peak duration were obtained by using a custom MATLAB code as we previously described [11]. Briefly, force calculations were performed based on data collected from the bright-field videos to assess tissue diameter as well as red fluorescence videos to collect wire displacement during tissue contraction as we previously described [11]. Displacement of fluorescent polymer wires as a result of cardiac tissue contraction was recorded at a frame rate of 100 frames s^−1^ using the fluorescence microscope under a 10× objective in the Texas Red channel (*λ*_ex_ = 596 nm, *λ*_em_ = 620 nm) to monitor spontaneous beating and stimulated beating of tissues under electrical stimulation at a voltage of two times the ET at 1 Hz, separately. The videos were then converted to stacks of still frames from which the wire displacement at the center was measured. The maximum (at the maximum contraction) and minimum (at the relaxed state) wire deflections were converted to force measurements, by using the calibration curves from force–displacement measurement by different customized tips listed above. The average tissue width (diameter) and width of the tissue wrapped around the polymer wire were measured from still frames of the 4X bright field video of the tissue in the relaxed position. The active force of the tissue is defined here as the difference between the maximum (total) force and the force measured at the minimum wire displacement, which we define as passive tension.

### 2.13. Immunostaining

The cardiac tissues were fixed with 4% paraformaldehyde (Sigma-Aldrich), permeabilized with 0.1% Triton X-100 (Alfa Aesar) in PBS and blocked with 5% goat serum in PBS. Tissues were then immunostained with mouse anti-cardiac troponin T (TNT) (Invitrogen; Catalogue number: MA5-12 960; 1:200), followed by goat anti-mouse-Alexa Fluor 647 (Invitrogen; Catalogue number: A-21 037; 1:400). Phalloidin-Alexa Fluor 488 (Invitrogen; Catalogue number: A-12 379; 1:200) was applied to stain F-actin fibers. Confocal fluorescence microscopy images were captured with an Olympus FluoView 1000 laser scanning confocal microscope.

### 2.14. Extracellular potential recording

The electrode pads on the edges of the conductive polymer micropillars (200 *µ*m) were connected to a custom PCB with electrophysiological amplification and digitization circuits (Intan RHS2116, Intan Technologies). The board was connected to a digital headstage controller (RHS Stim/Recording Controller, Intan Technologies). After the sample treatment with 2,3-butanedione monoxime (BDM), a myosin inhibitor [49] to minimize motion artifacts (1 h, in an incubator), a standard procedure in cardiac electrophysiology. The extracellular potential recording was performed with one of the CP pillar electrodes in the array as the reference electrode. The recording was performed using a sampling rate of 30 kHz. The signal was band-pass filtered at 1 Hz–5 kHz. A biphasic pulse, with a pulse width of 200 *µ*s and a current of 500 *µ*A, was applied to the device to stimulate the tissue at 1 Hz. The raw data was acquired by RHX data Intan acquisition software (Intan Technologies).

### 2.15. Electrical data analysis

The raw data were filtered using a Butterworth notch filter from MATLAB. The beat frequency was calculated using the fast Fourier transform function. The voltage–time curves were smoothed using Savitzky–Golay filtering or sgolayfilt function, to avoid interference with the baseline noise. A customized MATLAB code with the find-peaks function was used. The distance between peaks was calculated. The amplitude of the extracellular potentials was calculated from the heights of the positive and negative peaks. To measure the signal-to-noise ratio (SNR), the square of the field potential amplitude was divided by the square of the peak-to-peak amplitude of the baseline noise.

### 2.16. Statistical analysis

Statistical analysis was performed using Prism 9.0. Differences between experimental groups were analyzed by one-way ANOVA (more than two groups) or *t*-test (among two groups). The normality test (Shapiro-Wilk) was used for one-way ANOVA. Statistical significance was set at *p* < 0.05 and indicated in figures as * *p* < 0.05, ** *p* < 0.01, *** *p* < 0.001, **** *p* < 0.0001.

## 3. Results

### 3.1. Heart-on-a-chip platform with embedded 3D microelectrodes and 3D printed elastomeric microwires

The heart-on-a-chip platform (figure 1) consists of photolithographically patterned Au microelectodes with printed 3D PEDOT:PSS micropillars for extracellular field potential recording and elastomeric microwires as displacement sensors to measure the contractile force (figures 1(a), S1(a) and (b)). A pair of carbon electrodes is integrated to provide a capability for continuous, long-term, external electrical field stimulation.

Microfabricating high-aspect-ratio microstructures from conducting polymers is challenging, due to their low concentration in solution and the difficulty in accurately controlling the solvent evaporation. Here, a simple and fast microfabrication process was implemented to produce the 3D MEAs via meniscus-guided direct writing (figures 1(b) and (c)) according to a method we advanced previously for neural tissues [35, 41]. By using crosslinking agents and adjusting the fabrication parameters (i.e. nozzle diameter, PEDOT:PSS ink formulation, extrusion speed, temperature and humidity), the diameter of the microelectrodes was precisely controlled. The height was controlled by terminating the direct writing via a quick withdrawal of the printhead. Of note, due to the ‘soft’ contact and the tuned mechanical properties of fabricated PEDOT:PSS, the 3D printing method was capable of fabricating high-aspect-ratio micropillars. The soft and flexible micropillars were expected to yield to the contraction and relaxation of CMs (movie S1) and to embed into the tissue, enabling long-term culture and high-fidelity recording (figures 1(g) and (h)). For electrophysiological recording, the device was connected to a standard multichannel electrophysiological system via a PCB (figure S1(a)).

The need for manual production and manipulation of elastomeric polymer wires in our previous Biowire II platform [11] strongly motivated the development of microwire materials amenable to automated production, such as 3D printing (figures 1(d) and (e)), movie S3, figure S2). To achieve this, the nanocomposite ink was prepared from a TPE and core–shell QDs. Highly flexible nanocomposite TPE/QD microwires, designed to serve as both anchor points for the tissue and a displacement sensor to monitor the contractile behavior of the cardiac tissues, were directly 3D printed via a commercially available extrusion printer on both sides of the microwell on the bottom of the chamber, through a micronozzle (diameter of 60 *µ*m) (figure 1(d)). A cardiac tissue, based on iPSC-derived CMs and fibroblasts, wraps around the nanocomposite microwires (figure 1(f)), bending the wire with each contraction (figure 1(i)). The fluorescence of the QDs enabled *in situ* repeated measurement of the displacement of the wires via a standard fluorescence microscope and subsequent calculation of the contractile force of the iPSC-based cardiac tissue anchored to the nanocomposite wires, via force-displacement calibration curves (figure 1(j)).

### 3.2. Electrochemical and mechanical characterization of 3D microelectrodes

PEDOT:PSS microelectrodes’ height optimization (figures S3(a)–(c)) showed that the height of 250 ± 10 *µ*m and diameter: 5 ± 0.5 *µ*m ensures high enough electrodes’ protrusion into the tissue (movie S1). The high aspect ratio of 50 enables the electrode embedding into the tissue without obstruction to the beating, when the tissue is suspended above the hard substrate (movie S1). In regard to the vertical angle of the pillars, a small variation of about ±5°, among the micropillars was observed.

**Figure 2.**
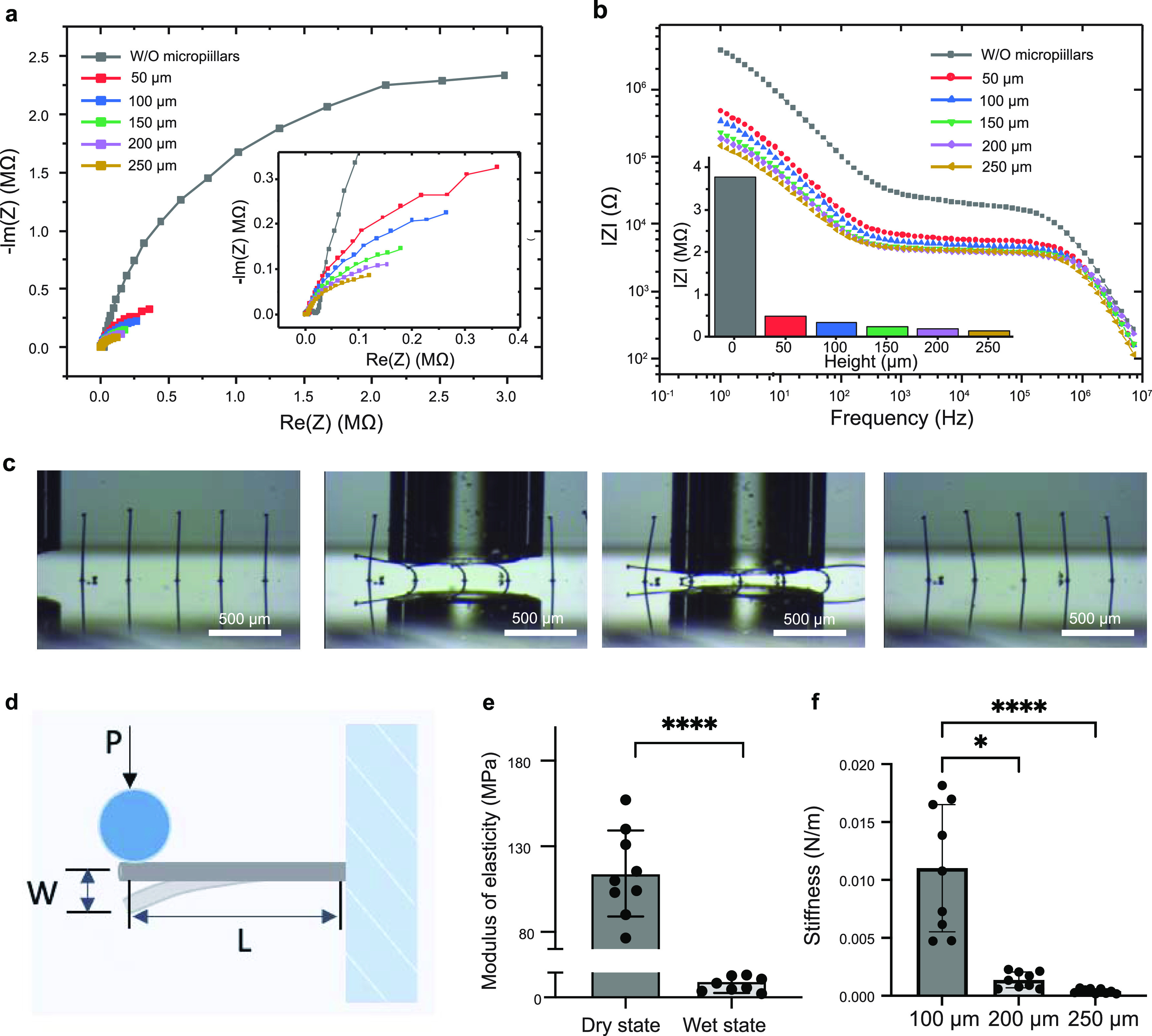
Three-dimensional-printed micropillar electrodes have tunable height, electrochemical properties and elasticity. (a) and (b) Electrochemical characterization of poly(3,4-ethylenedioxythiophene) (PEDOT) pillars of different heights, ranging from 50 *μ*m to 250 *μ*m, and PEDOT film without pillars. (a) Electrochemical impedance spectra (EIS), presented in the Nyquist plot, with the insert showing the high-frequency segment. (b) EIS presented in Bode plots. The impedance value at 1 Hz, relevant for the contractile behavior of cardiac tissue, is presented in the histogram insert. EIS were obtained in the presence of 5 mm Fe(CN)_6_^3-/4-^ in PBS. (c) Time-lapse images of CP micropillar displacement bent by a probe, showing reversible loading–unloading behavior and high flexibility. The gaps between the capillary and the substrate were 100 *µ*m and 50 *µ*m, respectively. Scale bar, 500 *µ*m. (d) Schematic of micropillar bending with the parameters for calculation of elastic modulus, where *L* is the CP micropillar length (measured from the bottom of the micropillar to the contact point), *P* is point load applied on the micropillar and *W* is the vertical displacement of the pillar at the contact point between the circular probe and the micropillar. (e) Elastic modulus of the micropillars in both dry and wet states. *n* = 9 (*t*-test, data shown as average ± s.d. **** indicates *p* < 0.0001). (f) The stiffness of the micropillars of heights of 100 *μ*m, 200 *μ*m and 250 *μ*m, in the wet state. *n* = 9 (One-way ANOVA, data shown as average ± s.d. * indicates *p* < 0.05, **** indicates *p* < 0.0001).

As shown by the cyclic voltammograms (CVs) (figure S3(d)), the reduction peak of the 3D PEDOT:PSS microelectrodes appeared at around −0.4 V in the negative scan, while the reverse process was characterized by a broad oxidative wave where the PEDOT oxidation process overlapped with the capacitive charging of the electrodes [50, 51]. The high CV currents suggest good conductivity of the pillars at potentials exceeding −0.3 V, providing a sufficient working range for the microelectrodes to record the cardiac field potentials which are generally observed within −150 *µ*V to +100 *µ*V. A positive correlation was found between voltammetric currents (between −0.3 V to +0.6 V) and micropillar height, which could be attributed to the increased mass and higher surface area of the pillars. For example, during the positive scan, the current (at 0 V) increased from 2.6 nA without pillars to 17.3 nA with 50 *µ*m pillars and further to 38.5 nA with 250 *µ*m pillars (figure S3(d)). This is in agreement with the trend in the electrodes’ impedance, where longer microelectrodes showed decreased impedance (figure 2(a)). Remarkably, the introduction of 3D microelectrodes significantly decreased the impedance and increased the electrochemical activity compared to the Au electrodes with electropolymerized PEDOT:PSS thin films (‘W/O micropillars’, figures 2(a) and S3(d)). For example, at 1 Hz, close to the beating rate of cardiac tissues, the thin film electrodes (W/O micropillars) showed ∼6.5 times higher impedance than the 3D microelectrodes with 50 *µ*m high pillars (figure 2(b)).

Micropillars’ flexibility was demonstrated by mechanical bending using a glass capillary (movie S2, figure 2(c)), which showed reversible loading-unloading behavior of the pillars with high flexibility. The micropillars are stabilized on the surface by the thin layer PEDOT:PSS that is first coated on top of the Au electrodes with the dual purpose: (i) to decrease the contact resistance and (ii) to enhance the bonding between the pillars and Au electrodes.

The force–displacement curves of the micropillars obtained by microscale mechanical testing (figures 2(d)–(f) and S4) in both dry (air, room temperature) and wet states (fully hydrated with culture medium) reproducibly displayed linear elastic behavior (figure S4). After incubation in the culture medium, the elastic modulus of the micropillars decreased dramatically from ∼118 MPa to around 310 kPa (figure 2(e)). This is due to the strong water absorption ability of PSS that turns the initially stiff dry pillars into a soft hydrogel. The stiffness variation with micropillar height (100, 200 and 250 *µ*m) (figure 2(f)) enabled tuning of structural properties to match those of the cardiac tissue, resulting in a heart-on-a-chip device with microelectrodes that did not impede the natural tissue motion (movie S1).

### 3.3. Tracking force *via* displacement of 3D printed fluorescent nanocomposite microwires

**Figure 3.**
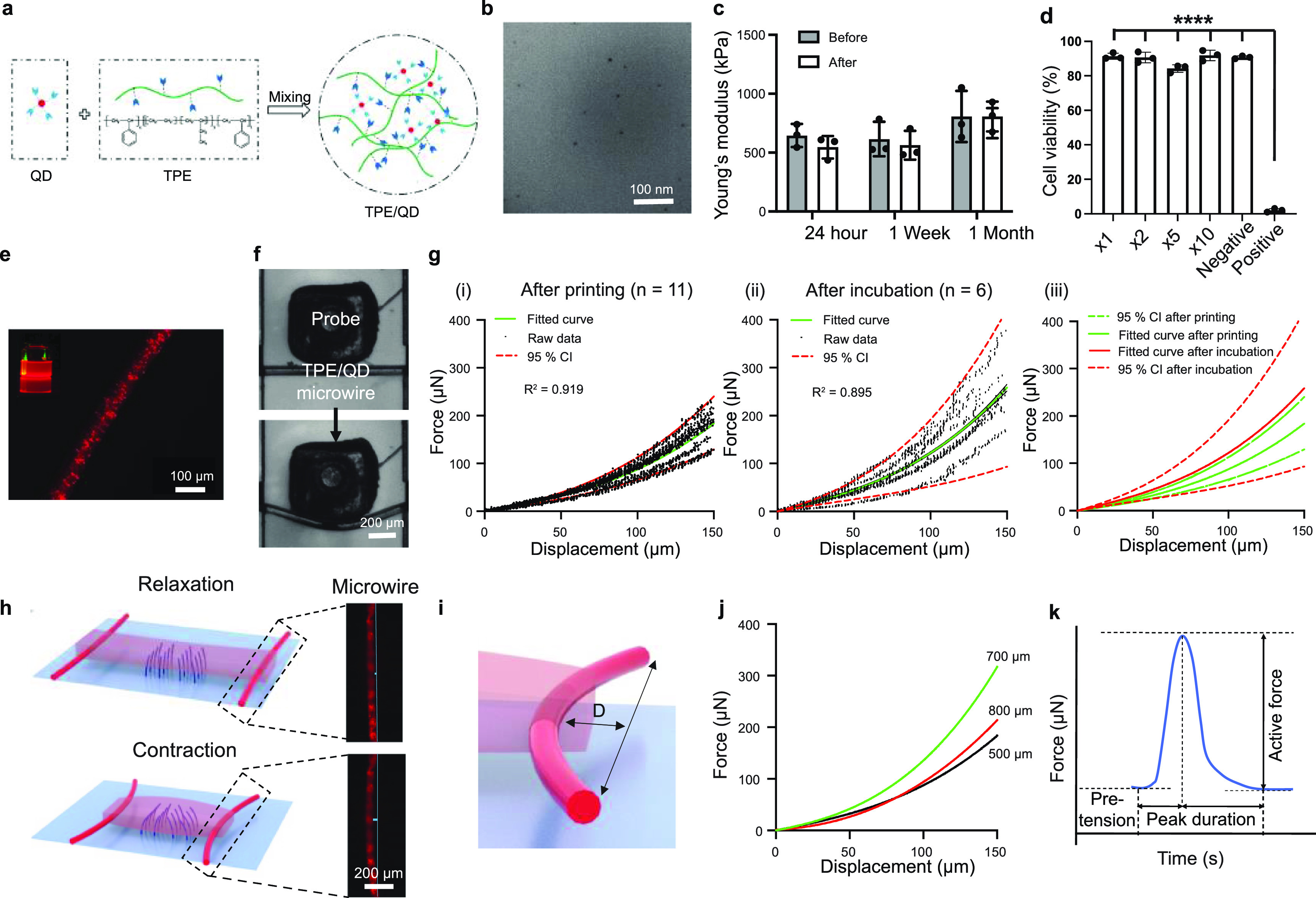
TPE/QD nanocomposites are stable, biocompatible, 3D-printable and highly flexible for non-invasive and *in situ* force sensing applications. (a) Schematic of nanocomposite ink preparation consisting of quantum dots (QD) and thermoplastic elastomer (TPE). (b) A transmission electron microscopy image of the TPE/QD nanocomposite shows that the quantum dot (QD) nanoparticles are well-dispersed in the thermoplastic elastomer (TPE). Scale bar: 100 nm. (c) Young’s modulus of TPE/QD nanocomposite slab before and after immersion in culture medium for 24 h, 1 week and 1 month, at room temperature. *n* = 3, data shown as average ± s.d. (d) Cardiac fibroblast viability, measured by lactate dehydrogenase (LDH) release, after culture for 24 h in the medium exposed to the TPE/QD nanocomposite material for 1 month. Negative controls were treated with the culture medium that had not been exposed to the nanocomposite. The positive controls were treated with the culture medium containing 1% Triton-X. Data presented as mean ± s.d., *n* = 3, ****p < 0.0001, one-way ANOVA. (e) A representative fluorescence microscopy image of the 3D printed nanocomposite microwire doped with CdSe/ZnS core–shell type QD nanoparticles with fluorescence maximum at *λ*_em_ 630 nm. Scale bar: 100 *μ*m. (f) Microscale mechanical testing of TPE/QD microwires. The microwire bent by a 500 *μ*m diameter customized probe. Scale bar: 200 *μ*m. (g) Force–displacement data points and fitted curves for TPE/QD nanocomposite microwires, (i) immediately after 3D printing, (ii) after 6-week incubation in culture media, and (iii) comparison of the two conditions to illustrate stability, *n* ⩾ 6. The fitted curve and *R* ^2^ values were presented and analyzed by Prism 9.0. (h) Schematics illustrating micropillars bending with the contraction and relaxation of the cardiac microtissue, with fluorescent microscopy images of TPE/QD microwire in relaxed and contracted states. Scale bar: 200 *μ*m. (i) Schematic illustration showing the deflection of the nanocomposite microwire due to the tissue contraction. D represents the displacement of the microwire bent under the contraction. (j) The calibration curves from different customized probes (dia. 500, 700 and 800 *μ*m), were generated from experimental data that were fit to a third-degree polynomial equation. (k) The quantification of contraction, relaxation and the total duration time (e.g. active force, pre-tension, peak duration) of the engineered tissue in the device can be evaluated from the deflection of the microwire using the calibration curves.

The nanocomposite microwires (61 ± 4 *µ*m), composed of TPE styrene-ethylene/butylene–styrene block copolymer and core–shell CdSe/ZnS QDs (QDs/TPE microwires) (figure 3(a)), in the heart-on-a-chip device serve an important dual purpose: 1) to stably anchor the tissue and 2) to enable displacement tracking for contractile force evaluation. The QDs/TPE nanocomposite can be extruded at elevated temperatures to enable automated production without the need for a sacrificial layer (movie S3), which is not possible with the elastomeric polyesters we used in Biowire II technology before [11]. QDs stabilized with octadecylamine ligands were used to match the hydrophobic nature of the TPE polymers, resulting in an appropriate polymer-ligand interface and good nanocomposite dispersion (figure 3(b)). This approach may also allow for customization of the color of the wire based on the color of the QDs; in contrast to our previously used poly(octamethylene maleate (anhydride) citrate) (POMAC) wires that relied on blue autofluorescence exclusively [11]. Therefore, we demonstrate the utilization of the red microwires based on 6 nm core–shell CdSe/ZnS QDs with a maximum fluorescence *λ*_em_ = 630 nm (figure 3(e)). The Young’s modulus of the TPE/QD nanocomposite (as investigated in a dog-bone slab format) remained stable even after a month of immersion in the culture medium (figures 3(c) and S5), demonstrating TPE/QD composite stability, important for longer-term cell cultivation studies. Biocompatibility of the TPE/QD nanocomposite is critical for long-term heart-on-chip applications. No decrease in the fibroblast cell viability after one month of the cell culture in the presence of the nanocomposite film was identified, compared with the culture media alone as the control (figure 3(d)).

After demonstrating mechanical stability of the nanocomposite slab upon long-term immersion in the culture medium, it was important to demonstrate such stability for the thin microwires made from the TPE/QD material. The force-displacement curves of the microwires in the device were unchanged after 6 weeks of incubation in culture media in the device indicating stability (figure 3(f) and (g)).

Calibration curves for tracking contraction forces are generated with probes of different sizes to mimic the size of the tissue (500, 700, 800 *μ*m) on the microwire. At low displacements, the microwire deflection exhibits a linear behavior with increasing force, whereas at high displacements, geometric non-linearities in the beam deformation cause the non-linear force-displacement curve, which is especially evident as the indenter probe size increases (figures 3(j) and S7).

The initially cell-seeded gel suspension remodels to a cylindrical tissue that is anchored to the TPE/QD wires and lifts up from the bottom of the substrate during the first week after cell seeding (figures 3(h), S1(c) and (d)). The TPE/QD micowires underwent the bending cycles as the cardiac tissue contracted (figure S6). By measuring the displacement at the wire center, where the displacement is the largest, via a conventional fluorescence microscope, one can evaluate the contraction force at timed intervals using previously obtained force–displacement calibration curves (figures 3(h)–(k) and S7). Importantly, most of the microwire displacement by the tissue contraction occurs up to ∼25 *µ*m, i.e. when the three calibration curves largely overlap (figures 3(j) and S7). Additionally, when experimental error is taken into account (figure S7(d)) the ranges of the force-displacement measurements in the calibration curves also overlap at larger deformations of the microwire.

### 3.4. Cardiac tissue formation and characterization

**Figure 4.**
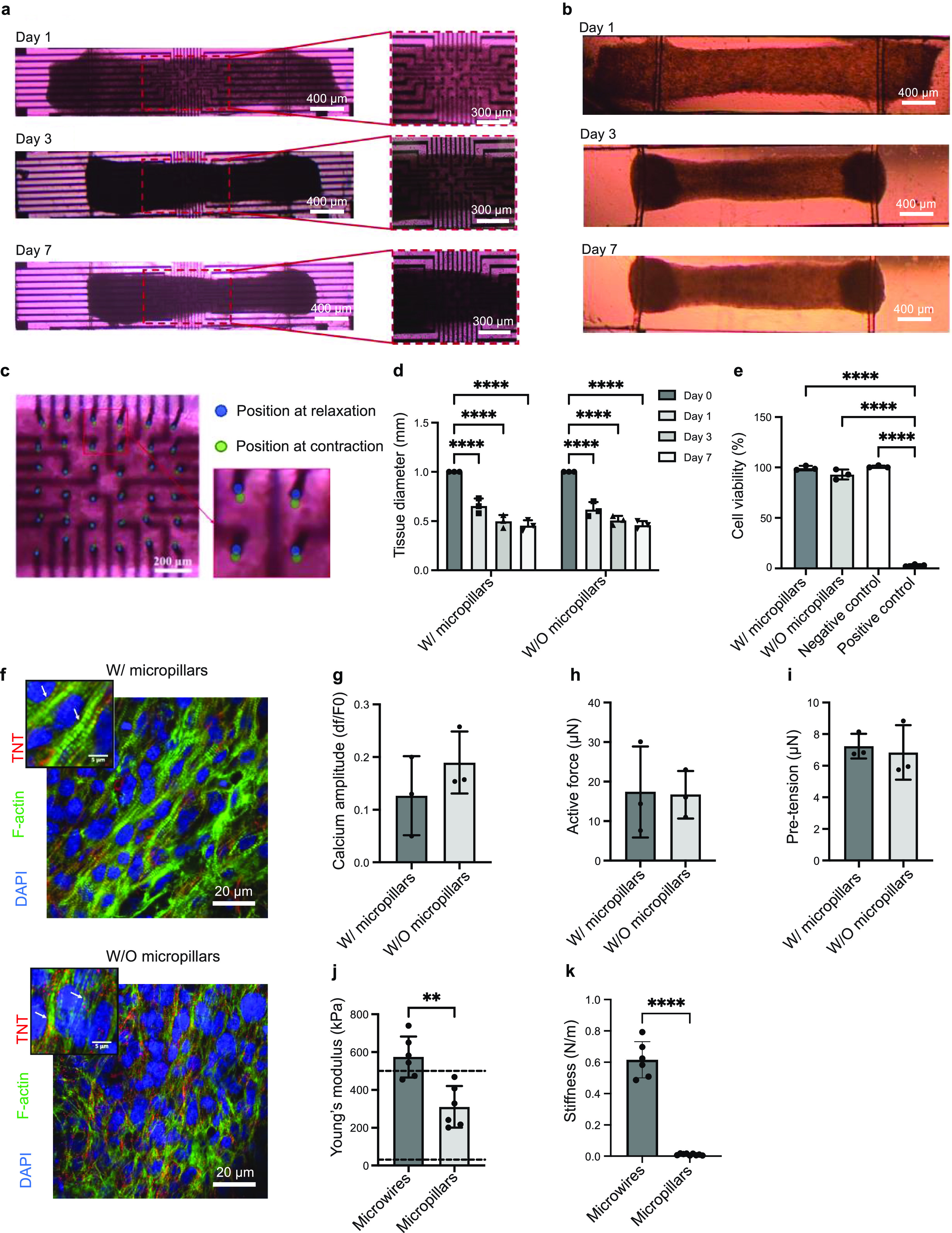
Micropillar electrodes allow for the formation of cardiac tissue and unobstructed beating. (a) Representative cardiac microtissues, showing the tissue compaction in the platforms with or b) without CP micropillars for a duration of 7 d after cell seeding. Scale bar: 400 and 300 *μ*m. (c) Optical images show the flexibility of the micropillars in the relaxation and contraction states of the tissues. Scale bar: 200 *μ*m. (d) Diameters of the tissues formed in the platforms with or without CP micropillars, at day 0, 1, 3, and 7 after cell seeding. (one-way ANOVA, *n* = 3, data shown as average ± s.d., **** indicates *p* < 0.0001). (e) Cell viability, measured by lactate dehydrogenase (LDH) release into the culture medium of microtissues cultivated for two weeks in the micropillar-based MEA device compared with that in the device without CP micropillars (one-way ANOVA, *n* = 3, data shown as average ± s.d., *** indicates *p* < 0.001, **** indicates *p* < 0.0001). (f) Confocal images of representative cardiac microtissue cultivated for five weeks in the platform with and without CP micropillars, immunostained for nucleus (DAPI), sarcomeric F-actin in green and cardiac troponin-T (TNT) in red. Scale bar: 20 *μ*m. (g) Calcium amplitude, (h) active force and (i) pre-tension of cardiac microtissues cultivated for one week in the platform with and without micropillars, paced at 1 Hz, *n* = 3, data shown as average ± s.d. (j) Young’s moduli of TPE/QD microwires and CP micropillars in the wet state, *n* = 6, data are shown as average ± s.d., ** indicates *p* < 0.01 Dashed lines indicate lower and upper range for native myocardium [52, 53, 60, 61]. (k) Maximum stiffness of TPE/QD microwires and CP micropillars in the wet state, *n* = 6, data are shown as average ± s.d., **** indicates *p* < 0.0001.

The 3D cardiac tissue created in the platform with 250 *μ*m high PEDOT:PSS micropillars eventually took on a cylindrical form (figure 4(a)). Similar levels of tissue compaction were observed in the tissues cultured in the device without 3D PEDOT:PSS microelectrodes (figure 4(b)), indicating that the soft conductive microelectrodes did not impede the tissue remodeling process (figure 4(d)), which is critical for the formation of trabeculae-like structure. During tissue compaction, the tractional forces displaced the outermost microelectrodes in the array by ∼20 *μ*m (figure S8). These data demonstrate that due to the low Young’s modulus of the micropillars, these soft structures do not deform the tissue during compaction. Instead, the tissue slightly displaces the pillar.

Importantly, PEDOT:PSS microelectrodes reversibly and repeatedly bent with the tissue contraction (figure 4(c) and movie S1), and reached a maximum displacement of ∼22 *µ*m, as measured during the contraction–relaxation cycles.

The biocompatibility of the microelectrode-based device was investigated by evaluating the cardiac tissue viability after 2 weeks of tissue culture in the device. The cells viability, as assessed by the LDH release, remained high and unaffected, regardless of the presence of the micropillars (figure 4(e)).

The electrical excitability of the tissue was enhanced during culture in the device, as evidenced by a significant decrease in the ET and a gradual increase in MCR (figure S9) over 5 weeks in culture. The tissues cultivated in the devices with or without micropillars exhibited well-aligned sarcomere structures, as demonstrated by cardiac TNT and F-actin immunostaining after 5 weeks in culture (figure 4(f)).

In CMs, the development of active force follows a calcium transient, motivating the inclusion of Ca^2+^ transient measurements in the new heart-on-a-chip device (movie S4, movie S5). Those measurements were realized through the use of non-ratiometric Ca^2+^ dye, Fluo 4, to enable *in situ* multi-parametric recordings. Importantly, there were no significant differences in calcium amplitude, active force and the pre-tension of the cardiac tissues cultured in the devices with or without micropillars, suggesting that the micropillars did not negatively impact the functional development of the tissues (figures 4(g)**–(**i)).

These advantageous characteristics of the platform were enabled by matching the mechanical properties in the wet state of the displacement-sensing TPE/QD microwires and the filed potential-sensing flexible 3D PEDOT:PSS microelectrodes to that of the cardiac tissue (maximum value of 500 kPa) [52, 53] (figure 4(j)), which is also important for physiological tissue assembly. Conveniently, TPE/QD nanocomposite wires were stiff enough (0.004 N m^−1^) to enable the nanocomposite wires to act as an anchor for tissue formation, while the stiffness of 3D conductive PEDOT:PSS microelectrodes was low enough to allow them to move along with the tissue beating motion (figure 4(k)). Importantly, the yield of microwires after 3D printing was essentially 100% and not a single wire broke during the course of the experiment, i.e. up to 5 weeks of cultivation.

### 3.5. Multi-parametric recording in the heart-on-a-chip device upon drug addition

**Figure 5.**
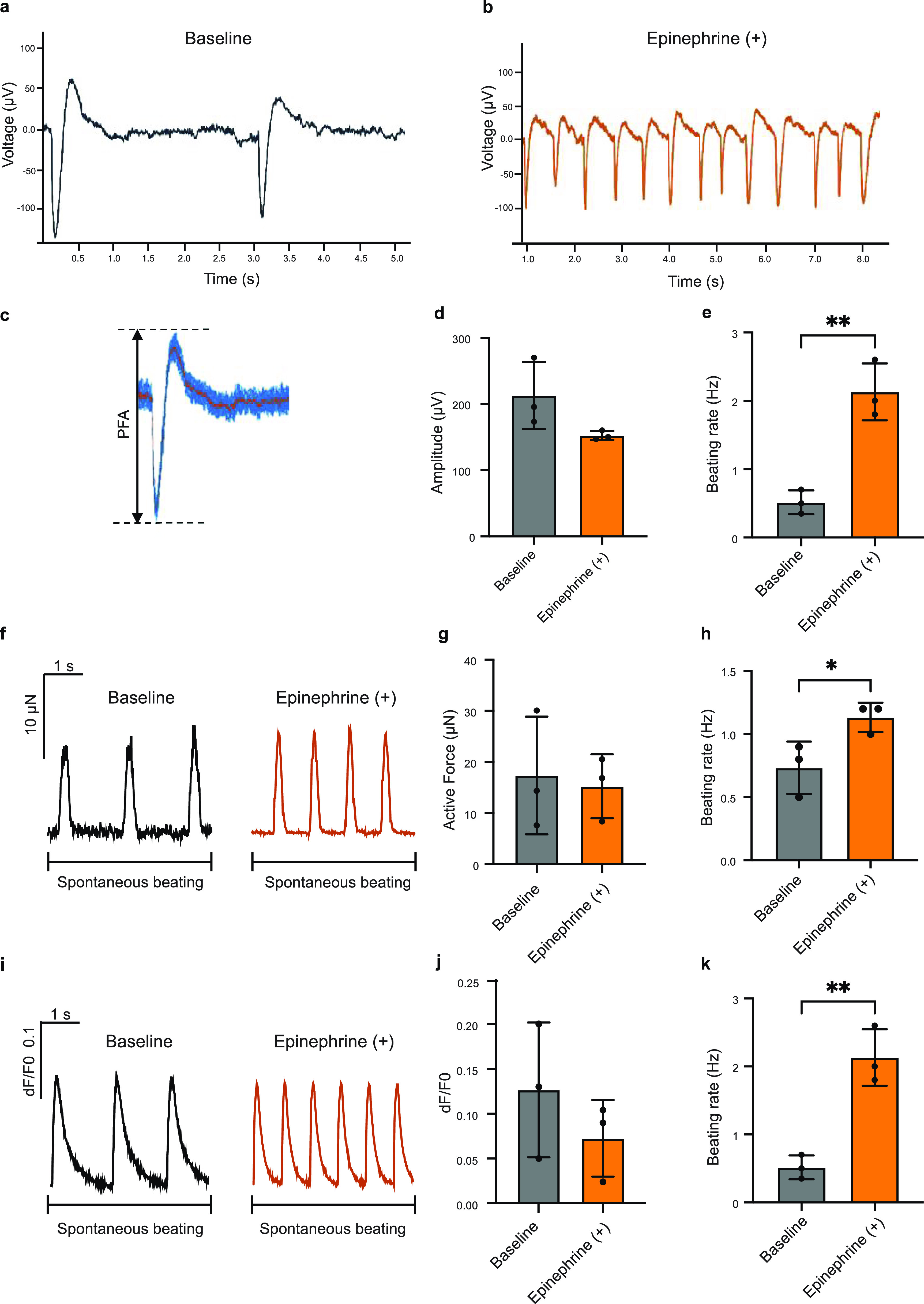
The heart-on-a-chip with 3D-printed microelectrodes and nanocomposite force sensors enables high-content measurement of extracellular field potentials and contractile forces, along with Ca^2+^ transients. Typical extracellular potential traces of the cardiac microtissues from the microelectrode-based device under spontaneous beating (a) before and (b) after epinephrine treatment. (c) Schematic figure of a peak, showing (d) field potential amplitude (FPA) from the extracellular potential recording of the tissue in the microelectrode-based device under spontaneous beating. (e) Quantified beating rate of the tissues before and after epinephrine treatment (*t*-test, *n* = 3, data shown as mean ± s.d. **p < 0.01). (f) Representative force traces of spontaneously beating cardiac microtissues after one week in culture, before vs. after epinephrine treatment. A time interval of 4 s is shown. Quantification of (g) active force and (h) beating rate of tissues before and after epinephrine treatment (*t*-test, *n* = 3, data shown as mean ± s.d., *p < 0.05). (i) Representative Ca^2+^ transient traces of cardiac tissues loaded with a Ca^2+^ dye (Fluo-4) under spontaneous beating after one week in culture before and after epinephrine treatment. A time interval of 4 s is shown. Quantification of (j) calcium amplitude, (k) beating rate of tissues before and after epinephrine treatment (*t*-test, *n* = 3, data shown as mean ± s.d., **p < 0.01).

To demonstrate the capacity of the platform to capture physiological responses of heart tissue, epinephrine was applied to induce a positive chronotropic response [54]. The platform successfully monitored the extracellular potentials of the cardiac tissues (figures 5(a)–(e) and movie S6), with the peak-to-peak noise measuring at ∼20 *μ*V and the SNR falling in the range of 33–35 both with and without epinephrine treatment. In this case, there were no significant differences in the signal amplitude between the drug treated and untreated samples (figure 5(d)). The spontaneous firing rate from the extracellular recordings increased, as expected, upon epinephrine addition (figure 5(e)). Recording from multiple electrodes within a single tissue was possible without and with the drug (figure S10). Motion artifacts were successfully eliminated through the use of BDM (figures S10 and S11).

The TPE/QD microwires effectively enumerated the spontaneous contractile dynamics in the tissues without and with epinephrine (figure 5(f), movie S4), demonstrating a comparable force amplitude (figure 5(g)) and a significantly higher spontaneous beating rate (figure 5(h)) upon drug addition, as expected. Consistently, peak duration, time from peak and time to peak all decreased upon epinephrine addition (figure S12).

Following epinephrine treatment, there were no significant differences in Ca^2+^ transient amplitude (figures 5(i) and (j)), consistent with the active force measurements, and the positive chronotropic effects were noted by the increase in the spontaneous Ca^2+^ transient rate as expected (figure 5(k)).

Additionally, our heart-on-a-chip platform allowed the multi-parametric recordings of the contraction force and Ca^2+^ measurements under pacing at 1 Hz, achieved through a pair of the embedded carbon electrodes (figures S13 and S14).

## 4. Discussion

Complex, mechanically and electrically active tissues, such as the heart muscle, require comprehensive profiling of a multitude of functional properties to appropriately benchmark disease phenotypes or the effects of test molecules. Rapidly developing systems for replication of human biology *in vitro* [3, 11, 21, 55–58], motivate the development of new approaches for the integration of multifaceted biosensors into 3D tissues and organ-on-a-chip devices for online functional assessments [59]. Heart-on-a-chip technologies, capable of both delivering controlled electrical stimuli and obtaining high-content recordings, are needed for comprehensive simultaneous profiling of multiple functional properties.

The heart-on-a-chip platform described here uniquely integrated 3D PEDOT:PSS soft micropillar electrodes and 3D-printed microwire displacement sensors to facilitate multifaceted functional *in situ* evaluation of cardiac tissue. This required appropriate consideration of the mechanical properties of the microwire and the microelectrode materials. The elastic modulus of the hydrated PEDOT:PSS micropillars (∼310 kPa) is similar to that of the native heart tissue (below 500 kPa) [52, 53, 60, 61], which is much lower than that of traditional electrode materials (range of GPa). The intrinsically swollen, water-rich nature of PEDOT:PSS hydrogel microelectrode also promotes the transport of chemical and biological molecules [62], thereby offering an extracellular matrix-like environment for tissue formation and growth. The mixed ionic and electronic conductivity of PEDOT:PSS is thought to further facilitate the effective coupling between the 3D microelectrode and the tissue.

Achieving a small diameter and high aspect ratio for the soft and flexible micropillars was challenging, but it was critical for sensor function with low impedance, which improved signal readout. The small diameter (5 ± 0.5 *μ*m) of the micropillars (smaller than the diameter of a single CM) was chosen to appropriately allow for electrical conduction of the cardiac tissue. Such a small size ensures that there are no interruptions in cell-to-cell contact, which is important for the function of the cardiac syncytium. The high aspect ratio also ensured that the electrodes can be embedded into the tissue, without anchoring the tissue to the underlying hard surface. The high aspect ratio of the micropillar electrodes means only a small force is required to bend the pillars, as cantilevers, from the top. The PEDOT:PSS microelectrodes were robust enough to remain stably connected to the gold interconnects at the MEA, despite cyclic displacement due to the cardiac tissue contraction for up to 5 weeks, retaining the capability to record electrical signals even after being immersed in culture media. The thin electropolymerized layer of PEDOT:PSS adheres well to the Au electrodes, likely via a combination of non-covalent van der Waals forces and chemisorption interactions between S from PEDOT and Au, to stabilize micropillar electrodes on the MEA. In addition, the PEDOT:PSS pillars’ bonding to the PEDOT thin layer is enhanced by the covalent cross-linking of the pillars by GOPS. The pillars always supported their own weight (at the aspect ratios investigated) and remained vertical when the cardiac tissue is cultured on the array. The pillars remained patent over 5 weeks of culture, without significant observable cell deposition on the electrodes.

Although extracellular field potentials differ between CM species (e.g. mouse *vs* human) [11, 63], and cardiac cell lines [64] *vs* stem cell-derived CMs [11], our data were consistent with other studies reporting the use of human iPSC derived CMs in novel devices capable of extracellular field potential recording [65].

Importantly, another set of integrated electrodes, two parallel carbon electrodes, was placed into the device to drive external field stimulation during cell culture, to promote the functional improvement of the tissue, as external electrical stimulation is known to be beneficial for cardiac tissue assembly [11, 66]. We delivered up to 12 V in biphasic pulses (100 *µ*s per phase, 200 *µ*s total duration) at 1 Hz through a micropillar electrode or through all 16 micropillar electrodes at once to induce point stimulation. The maximum current went up to 2 mA, although the typical stimulus was 500 *µ*A. Although the array successfully delivered the voltage, there was no measurable contraction of the tissue. Therefore, we decided to use the embedded parallel carbon electrodes instead (shown as dark grey rods in figure S2) to deliver field stimulation, since we were able to more reproducibly induce contraction in the tissue this way.

Our widely used Biowire II platform relies on two parallel POMAC wires to anchor the tissue and measure contractile properties [11, 67, 68]. Yet, POMAC is not amenable to automated and scalable fabrication methods since it is not thermosetting and its UV crosslinking time is too long for effective 3D printing. More fundamentally, displacement tracking in POMAC wires relies on autofluorescence, which is the highest in the blue channel, effectively limiting the number of wavelengths that can be used for detection.

This motivated us to develop a 3D flexible microwire as an optical force sensor based on semiconductor QDs dispersed in rapidly setting TPE amenable to 3D printing. Yet, the use of fluorophores in solid-state applications is typically more challenging than in solution-based applications, as high concentrations of fluorophores are needed in the solid state to enhance the photoluminescence intensity of the structure. This leads to an unfavorable effect of self-quenching, an issue we had to overcome in the current work. In the current setup, a low concentration (0.05 w/w%) of QDs in the nanocomposite was used to avoid the inner filter effect that drives self-quenching [69]. The described approach eliminated the manual insertion steps as in our previous studies [11, 67, 68], a requirement for increased production throughput of heart-on-a-chip devices and ultimately their widespread adoption. Importantly, the deformation of the microwire occurs as a result of contraction of the tissue and not the other way around (i.e. the microwires are not moved externally to move the tissue, but the paced tissue contraction causes displacement of the wire). The wires provide passive mechanical resistance, due to their Young’s modulus (574.14 ± 108.2 kPa) which matches that of the native cardiac tissue (a maximum value of 500 kPa) [52, 53].

The described platform is the first to enable integrated measurements of field potential, contractile force and Ca^2+^ transients from 3D trabecula-like human cardiac tissues, paving the way to high-content heart-on-a-chip devices.

## 5. Conclusions

Here, we engineered an instrumented heart-on-a-chip platform featuring soft, electrically conductive, micropillars as 3D microelectrodes for non-invasive and high-resolution *in situ* monitoring of electrophysiological signals, and elastic TPE/QD nanocomposite microwires as displacement sensors for real-time recording of cardiac tissue contractile properties, under both spontaneous and paced conditions. A separate pair of integrated carbon electrodes enabled continuous field stimulation. Mechanical interference of the electrodes with the cardiac tissue was minimal due to both the flexibility of the conductive polymer pillars and the high aspect ratio of the microelectrodes. The 3D printing approach used here facilitates customization to achieve tissue-like mechanical properties and sensor dimensions fit for the required functional readouts, thereby dramatically improving both data acquisition capability and the relevance of the system for biological studies.

## Data Availability

The data cannot be made publicly available upon publication because no suitable repository exists for hosting data in this field of study. The data that support the findings of this study are available upon reasonable request from the authors.

## References

[1] Zhang B, Korolj A, Lai B F L, Radisic M (2018). Advances in organ-on-a-chip engineering. Nat. Rev. Mater..

[2] Zhang B, Radisic M (2017). Organ-on-a-chip devices advance to market. Lab Chip.

[3] Bliley J M, Vermeer M C, Duffy R M, Batalov I, Kramer D, Tashman J W, Shiwarski D J, Lee A, Teplenin A S, Volkers L (2021). Dynamic loading of human engineered heart tissue enhances contractile function and drives a desmosome-linked disease phenotype. Sci. Trans. Med..

[4] Low L A, Mummery C, Berridge B R, Austin C P, Tagle D A (2021). Organs-on-chips: into the next decade. Nat. Rev. Drug Discovery.

[5] Wang X, Wang L, Dou W, Huang Z, Zhao Q, Malhi M, Maynes J T, Sun Y (2020). Electrical impedance-based contractile stress measurement of human iPSC-cardiomyocytes. Biosens. Bioelectron..

[6] Hildebrandt M R, Reuter M S, Wei W, Tayebi N, Liu J, Sharmin S, Mulder J, Lesperance L S, Brauer P M, Mok R S (2019). Precision health resource of control iPSC lines for versatile multilineage differentiation. Stem. Cell Rep..

[7] Spira M E, Hai A (2013). Multi-electrode array technologies for neuroscience and cardiology. Nat. Nanotechnol..

[8] Qian F, Huang C, Lin Y-D, Ivanovskaya A N, O’Hara T J, Booth R H, Creek C J, Enright H A, Soscia D A, Belle A M (2017). Simultaneous electrical recording of cardiac electrophysiology and contraction on chip. Lab Chip.

[9] Feric N T, Radisic M (2016). Maturing human pluripotent stem cell-derived cardiomyocytes in human engineered cardiac tissues. Adv. Drug Deliv. Rev..

[10] Zhao Y, Rafatian N, Wang E Y, Wu Q, Lai B F, Lu R X, Savoji H, Radisic M (2020). Towards chamber specific heart-on-a-chip for drug testing applications. Adv. Drug Deliv. Rev..

[11] Zhao Y, Rafatian N, Feric N T, Cox B J, Aschar-Sobbi R, Wang E Y, Aggarwal P, Zhang B, Conant G, Ronaldson-Bouchard K (2019). A platform for generation of chamber-specific cardiac tissues and disease modeling. Cell.

[12] Lee S, Serpooshan V, Tong X, Venkatraman S, Lee M, Lee J, Chirikian O, Wu J C, Wu S M, Yang F (2017). Contractile force generation by 3D hiPSC-derived cardiac tissues is enhanced by rapid establishment of cellular interconnection in matrix with muscle-mimicking stiffness. Biomaterials.

[13] Marsano A, Conficconi C, Lemme M, Occhetta P, Gaudiello E, Votta E, Cerino G, Redaelli A, Rasponi M (2016). Beating heart on a chip: a novel microfluidic platform to generate functional 3D cardiac microtissues. Lab Chip.

[14] Lu H F, Leong M F, Lim T C, Chua Y P, Lim J K, Du C, Wan A C (2017). Engineering a functional three-dimensional human cardiac tissue model for drug toxicity screening. Biofabrication.

[15] Hansen K J, Favreau J T, Gershlak J R, Laflamme M A, Albrecht D R, Gaudette G R (2017). Optical method to quantify mechanical contraction and calcium transients of human pluripotent stem cell-derived cardiomyocytes. Tissue Eng. C.

[16] Arslan U, Moruzzi A, Nowacka J, Mummery C, Eckardt D, Loskill P, Orlova V (2022). Microphysiological stem cell models of the human heart. Mater. Today Biol..

[17] Huebsch N, Loskill P, Mandegar M A, Marks N C, Sheehan A S, Ma Z, Mathur A, Nguyen T N, Yoo J C, Judge L M (2015). Automated video-based analysis of contractility and calcium flux in human-induced pluripotent stem cell-derived cardiomyocytes cultured over different spatial scales. Tissue Eng. C.

[18] Mathur A, Loskill P, Shao K, Huebsch N, Hong S, Marcus S G, Marks N, Mandegar M, Conklin B R, Lee L P (2015). Human iPSC-based cardiac microphysiological system for drug screening applications. Sci. Rep..

[19] Veldhuizen J, Cutts J, Brafman D A, Migrino R Q, Nikkhah M (2020). Engineering anisotropic human stem cell-derived three-dimensional cardiac tissue on-a-chip. Biomaterials.

[20] Hinson J T (2015). HEART DISEASE. Titin mutations in iPS cells define sarcomere insufficiency as a cause of dilated cardiomyopathy. Science.

[21] Wang G (2014). Modeling the mitochondrial cardiomyopathy of Barth syndrome with induced pluripotent stem cell and heart-on-chip technologies. Nat. Med..

[22] Feinberg A W, Feigel A, Shevkoplyas S S, Sheehy S, Whitesides G M, Parker K K (2007). Muscular thin films for building actuators and powering devices. Science.

[23] Sheehy S P, Pasqualini F, Grosberg A, Park S J, Aratyn-Schaus Y, Parker K K (2014). Quality metrics for stem cell-derived cardiac myocytes. Stem. Cell Rep..

[24] Sidorov V Y, Samson P C, Sidorova T N, Davidson J M, Lim C C, Wikswo J P (2017). I-wire heart-on-a-chip I: three-dimensional cardiac tissue constructs for physiology and pharmacology. Acta biomater..

[25] Feiner R, Engel L, Fleischer S, Malki M, Gal I, Shapira A, Shacham-Diamand Y, Dvir T (2016). Engineered hybrid cardiac patches with multifunctional electronics for online monitoring and regulation of tissue function. Nat. Mater..

[26] Kalmykov A, Huang C, Bliley J, Shiwarski D, Tashman J, Abdullah A, Rastogi S K, Shukla S, Mataev E, Feinberg A W (2019). Organ-on-e-chip: three-dimensional self-rolled biosensor array for electrical interrogations of human electrogenic spheroids. Sci. Adv..

[27] Smith A S, Choi E, Gray K, Macadangdang J, Ahn E H, Clark E C, Laflamme M A, Wu J C, Murry C E, Tung L (2019). NanoMEA: a tool for high-throughput, electrophysiological phenotyping of patterned excitable cells. Nano Lett..

[28] Choi J S, Smith A S, Williams N P, Matsubara T, Choi M, Kim J W, Kim H J, Choi S, Kim D H (2020). Nanopatterned Nafion microelectrode arrays for *in vitro* cardiac electrophysiology. Adv. Funct. Mater..

[29] Zhang F, Qu K-Y, Zhou B, Luo Y, Zhu Z, Pan D-J, Cui C, Zhu Y, Chen M-L, Huang N-P (2021). Design and fabrication of an integrated heart-on-a-chip platform for construction of cardiac tissue from human iPSC-derived cardiomyocytes and *in situ* evaluation of physiological function. Biosens. Bioelectron..

[30] Lind J U, Busbee T A, Valentine A D, Pasqualini F S, Yuan H, Yadid M, Park S-J, Kotikian A, Nesmith A P, Campbell P H (2017). Instrumented cardiac microphysiological devices via multimaterial three-dimensional printing. Nat. Mater..

[31] Grob L, Yamamoto H, Zips S, Rinklin P, Hirano‐Iwata A, Wolfrum B (2020). Printed 3D electrode arrays with micrometer‐scale lateral resolution for extracellular recording of action potentials. Adv. Mater. Technol..

[32] Xie C, Lin Z, Hanson L, Cui Y, Cui B (2012). Intracellular recording of action potentials by nanopillar electroporation. Nat. Nanotechnol..

[33] Lin Z C, Xie C, Osakada Y, Cui Y, Cui B (2014). Iridium oxide nanotube electrodes for sensitive and prolonged intracellular measurement of action potentials. Nat. Commun..

[34] Nick C, Quednau S, Sarwar R, Schlaak H, Thielemann C (2014). High aspect ratio gold nanopillars on microelectrodes for neural interfaces. Microsyst. Technol..

[35] Zhang P, Aydemir N, Alkaisi M, Williams D E, Travas-Sejdic J (2018). Direct writing and characterization of three-dimensional conducting polymer PEDOT arrays. ACS Appl. Mater. Interfaces.

[36] Liang Y, Offenhäusser A, Ingebrandt S, Mayer D (2021). PEDOT: PSS‐based bioelectronic devices for recording and modulation of electrophysiological and biochemical cell signals. Adv. Healthcare Mater..

[37] Yan L-P, Wen M-Y, Qin Y, Bi C X, Zhao Y, Fan W-T, Yan J, Huang W-H, Liu Y-L (2022). Soft electrodes for electrochemical and electrophysiological monitoring of beating cardiomyocytes. Angew. Chem., Int. Ed..

[38] Green R (2019). Elastic and conductive hydrogel electrodes. Nat. Biomed. Eng..

[39] Burnstine‐Townley A, Eshel Y, Amdursky N (2020). Conductive scaffolds for cardiac and neuronal tissue engineering: governing factors and mechanisms. Adv. Funct. Mater..

[40] Jimbo Y, Sasaki D, Ohya T, Lee S, Lee W, Hassani F A, Yokota T, Matsuura K, Umezu S, Shimizu T (2021). An organic transistor matrix for multipoint intracellular action potential recording. Proc. Natl Acad. Sci..

[41] Tomaskovic-Crook E (2019). Human neural tissues from neural stem cells using conductive biogel and printed polymer microelectrode arrays for 3D electrical stimulation. Adv. Healthcare Mater..

[42] Liu Y, McGuire A F, Lou H-Y, Li T L, Tok J B-H, Cui B, Bao Z (2018). Soft conductive micropillar electrode arrays for biologically relevant electrophysiological recording. Proc. Natl Acad. Sci..

[43] Lacour S P, Courtine G, Guck J (2016). Materials and technologies for soft implantable neuroprostheses. Nat. Rev. Mater..

[44] Laslau C, Williams D E, Kannan B, Travas-Sejdic J (2011). Scanned pipette techniques for the highly localized electrochemical fabrication and characterization of conducting polymer thin films, microspots, microribbons, and nanowires. Adv. Funct. Mater..

[45] Lian X, Zhang J, Azarin S M, Zhu K, Hazeltine L B, Bao X, Hsiao C, Kamp T J, Palecek S P (2013). Directed cardiomyocyte differentiation from human pluripotent stem cells by modulating Wnt/β-catenin signaling under fully defined conditions. Nat. Protocols.

[46] Yang L, Soonpaa M H, Adler E D, Roepke T K, Kattman S J, Kennedy M, Henckaerts E, Bonham K, Abbott G W, Linden R M (2008). Human cardiovascular progenitor cells develop from a KDR+ embryonic-stem-cell-derived population. Nature.

[47] Davenport Huyer L, Bannerman A D, Wang Y, Savoji H, Knee‐Walden E J, Brissenden A, Yee B, Shoaib M, Bobicki E, Amsden B G (2019). One‐pot synthesis of unsaturated polyester bioelastomer with controllable material curing for microscale designs. Adv. Healthcare Mater..

[48] Zhang B, Montgomery M, Chamberlain M D, Ogawa S, Korolj A, Pahnke A, Wells L A, Massé S, Kim J, Reis L (2016). Biodegradable scaffold with built-in vasculature for organ-on-a-chip engineering and direct surgical anastomosis. Nat. Mater..

[49] Sellin L, McArdle J (1994). Multiple effects of 2, 3‐butanedione monoxime. Pharmacol. Toxicol..

[50] Ouyang J, Xu Q, Chu C-W, Yang Y, Li G, Shinar J (2004). On the mechanism of conductivity enhancement in poly (3, 4-ethylenedioxythiophene): poly (styrene sulfonate) film through solvent treatment. Polymer.

[51] Xia Y, Ouyang J (2011). PEDOT: PSS films with significantly enhanced conductivities induced by preferential solvation with cosolvents and their application in polymer photovoltaic cells. J. Mater. Chem..

[52] Nagueh S F, Shah G, Wu Y, Torre-Amione G, King N M, Lahmers S, Witt C C, Becker K, Labeit S, Granzier H L (2004). Altered titin expression, myocardial stiffness, and left ventricular function in patients with dilated cardiomyopathy. Circulation.

[53] Omens J H (1998). Stress and strain as regulators of myocardial growth. Prog. Biophys. Mol. Biol..

[54] Noma A, Kotake H, Irisawa H (1980). Slow inward current and its role mediating the chronotropic effect of epinephrine in the rabbit sinoatrfal node. Pfluger’s Arch..

[55] Hinson J T, Chopra A, Nafissi N, Polacheck W J, Benson C C, Swist S, Gorham J, Yang L, Schafer S, Sheng C C (2015). Titin mutations in iPS cells define sarcomere insufficiency as a cause of dilated cardiomyopathy. Science.

[56] Wang E Y, Rafatian N, Zhao Y, Lee A, Lai B F L, Lu R X, Jekic D, Davenport Huyer L, Knee-Walden E J, Bhattacharya S (2019). Biowire model of interstitial and focal cardiac fibrosis. ACS Cent. Sci..

[57] Wang E Y, Kuzmanov U, Smith J B, Dou W, Rafatian N, Lai B F L, Lu R X Z, Wu Q, Yazbeck J, Zhang X-O (2021). An organ-on-a-chip model for pre-clinical drug evaluation in progressive non-genetic cardiomyopathy. J. Mol. Cell Cardiol..

[58] Tiburcy M, Hudson J E, Balfanz P, Schlick S, Meyer T, Chang Liao M-L, Levent E, Raad F, Zeidler S, Wingender E (2017). Defined engineered human myocardium with advanced maturation for applications in heart failure modeling and repair. Circulation.

[59] Reardon S (2015). Organs-on-chips’ go mainstream. Nat. News.

[60] Weis S M, Emery J L, Becker K D, McBride D J, Omens J H Jr, McCulloch A D (2000). Myocardial mechanics and collagen structure in the osteogenesis imperfecta murine (oim). Circ. Res..

[61] Coirault C, Samuel J L, Chemla D, Pourny J C, Lambert F, Marotte F, Lecarpentier Y (1998). Increased compliance in diaphragm muscle of the cardiomyopathic Syrian hamster. J. Appl. Physiol..

[62] Green R, Abidian M R (2015). Conducting polymers for neural prosthetic and neural interface applications. Adv. Mater..

[63] Halbach M, Egert U, Hescheler J, Banach K (2003). Estimation of action potential changes from field potential recordings in multicellular mouse cardiac myocyte cultures. Cell. Physiol. Biochem..

[64] Claycomb W C, Lanson N A, Jr Stallworth B S, Egeland D B, Delcarpio J B, Bahinski A, Izzo N J Jr (1998). HL-1 cells: a cardiac muscle cell line that contracts and retains phenotypic characteristics of the adult cardiomyocyte. Proc. Natl Acad. Sci. USA.

[65] Dou W (2022). A carbon-based biosensing platform for simultaneously measuring the contraction and electrophysiology of iPSC-cardiomyocyte monolayers. ACS Nano.

[66] Radisic M, Park H, Shing H, Consi T, Schoen F J, Langer R, Freed L E, Vunjak-Novakovic G (2004). Functional assembly of engineered myocardium by electrical stimulation of cardiac myocytes cultured on scaffolds. Proc. Natl Acad. Sci. USA.

[67] Zhao Y, Rafatian N, Wang E Y, Feric N T, Lai B F L, Knee-Walden E J, Backx P H, Radisic M (2019). Engineering microenvironment for human cardiac tissue assembly in heart-on-a-chip platform. Matrix Biol..

[68] Zhao Y, Wang E Y, Davenport L H, Liao Y, Yeager K, Vunjak-Novakovic G, Radisic M, Zhang B (2019). A multimaterial microphysiological platform enabled by rapid casting of elastic microwires. Adv. Healthcare Mater..

[69] Koc M A, Raja S N, Hanson L A, Nguyen S C, Borys N J, Powers A S, Wu S, Takano K, Swabeck J K, Olshansky J H (2017). Characterizing photon reabsorption in quantum dot-polymer composites for use as displacement sensors. ACS Nano.

